# Study of a Nonlinear Membrane Absorber Applied to 3D Acoustic Cavity for Low Frequency Broadband Noise Control

**DOI:** 10.3390/ma12071138

**Published:** 2019-04-08

**Authors:** Jianwang Shao, Tao Zeng, Xian Wu

**Affiliations:** School of Automotive Studies, Tongji University, Shanghai 201804, China; shaojianwang@tongji.edu.cn (J.S.); zrtao220@163.com (T.Z.)

**Keywords:** nonlinear membrane absorber, targeted energy transfer, 3D acoustic cavity, low frequency broadband noise, pre-stress

## Abstract

As a new approach to passive noise control in low frequency domain, the targeted energy transfer (TET) technique has been applied to the 3D fields of acoustics. The nonlinear membrane absorber based on the TET can reduce the low frequency noise inside the 3D acoustic cavity. The TET phenomenon inside the 3D acoustic cavity has firstly investigated by a two degrees-of-freedom (DOF) system, which is comprised by an acoustic mode and a nonlinear membrane without the pre-stress. In order to control the low frequency broadband noise inside 3D acoustic cavity and consider the influence of the pre-stress for the TET, a general model of the system with several acoustic modes of 3D acoustic cavity and one nonlinear membrane is built and studied in this paper. By using the harmonic balance method and the numerical method, the nonlinear normal modes and the forced responses are analyzed. Meanwhile, the influence of the pre-stress of the nonlinear membrane for the TET is investigated. The desired working zones of the nonlinear membrane absorber for the broadband noise are investigated. It can be helpful to design the nonlinear membrane according the dimension of 3D acoustic cavity to control the low frequency broadband noise.

## 1. Introduction

Many techniques, which include active noise control method [1] and vibration damping materials [2], are used to control noise inside a 3D enclosed cavity. The computer aided engineering (CAE) methods are applied to easily treat with interior acoustic problems, such as finite element method (FEM), boundary element method (BEM), and extended methods based on above CAE methods [3,4,5]. Recently, acoustic metamaterials are studied by the researchers to control sound waves, where a lot of progress has been made [6,7]. Meanwhile, there are many challenges in the practical implementation of acoustic metamaterials [8].

Since the concept of targeted energy transfer (TET) was proposed by Vakakis and Gendelman [9,10] in 2001, many studies have been made in view of application in the field of mechanical vibrations [11,12,13,14,15] by a purely nonlinear absorber called as nonlinear energy sink (NES). Meanwhile, in acoustic field, the TET phenomenon was firstly demonstrated inside one tube (1D acoustic system) by a nonlinear membrane NES [16]. The TET between the membrane and the tube for both free and forced oscillations was investigated [17,18]. Moreover, a loudspeaker working outside its linear regime was demonstrated that it could also be an efficient NES [19]. In these studies [16,17,18], the nonlinear membrane NES was used to reduce one acoustic mode of the tube standing for the linear system. Cote et al. [20] analyzed the TET phenomenon between the nonlinear membrane NES and two acoustic modes of the tube and observed the membrane could reduce the two resonance peaks, simultaneously.

In view of extending the application of the membrane NES in acoustic field, an acoustic cavity (3D acoustic system) was considered and the TET phenomenon was observed inside the acoustic cavity by the nonlinear membrane NES without considering the pre-stress of the membrane [21,22,23]. By analyzing the nonlinear normal modes (NNM) and the periodic forced responses of a two degrees-of-freedom (DOF) system comprised of one acoustic mode of cavity and a nonlinear membrane NES, the desired working zone for the membrane NES was defined and the two thresholds of the zone were also determined analytically and semi-analytically, respectively [21]. Based on these analytical results, the parametric analysis of the membrane was studied to reveal that the radius of the membrane affected mainly the desired working zone [22]. To extending the results obtained by the 2DOFs, the 3DOFs system with two nonlinear membranes and one acoustic mode was also investigated [23,24]. Two nonlinear membranes could enlarge the desired working zone of the NES.

In this paper, in order to control the low frequency broadband noise (20–200 Hz) inside 3D acoustic cavity and consider the influence of the pre-stress for the TET, a general model of the system with several acoustic modes of 3D acoustic cavity and one nonlinear membrane is firstly built. The influence of the pre-stress of the membrane for the TET is investigated. A multi-DOFs system comprised by a nonlinear membrane absorber and two acoustic modes or multi-acoustic modes are studied. The forced responses of the system are analyzed. The desired working zone and the value of the plateau for low frequency broadband noise of the nonlinear membrane absorber are investigated. Numerical simulations are finally preformed to validate the TET phenomenon of the system and the analytical results.

## 2. Description of the System

### 2.1. The Acoustic Cavity and the Membrane

**Figure 1:**
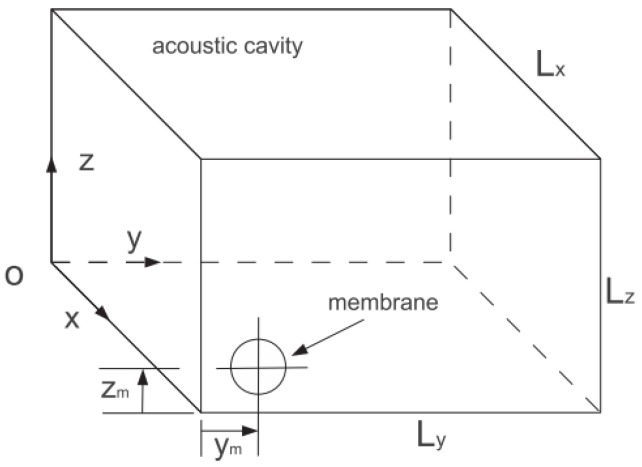
Schema of the acoustic cavity mounted a membrane.

The schema of the system in this paper is shown in Figure 1, which is comprised of an acoustic medium inside a parallelepiped cavity and a thin viscoelastic membrane that is mounted on one wall of the cavity. The dimensions of the acoustic cavity are $L_{x}$, $L_{y}$ and $L_{z}$ and the position of the membrane is marked as ($x_{m}$, $y_{m}$, $z_{m}$) ($x_{m}=L_{x}$).

In order to build a general model, the modes of 3D acoustic cavity between the frequency band 20–200 Hz and one nonlinear membrane are, respectively, considered as the linear system with multi-DOFs and the NES. We assume that six walls of the acoustic cavity shown in Figure 1 are rigid. Thus, the mode shape of the cavity $P_{lmn}$ is defined as following:

(1) $$P_{lmn}=\cos\left(\frac{l\pi x}{L_{x}}\right)\cos\left(\frac{m\pi y}{L_{y}}\right)\cos\left(\frac{n\pi z}{L_{z}}\right)$$

In this paper, we assume that the modes between the frequency band 20–200 Hz of the cavity are separated in frequency, and we focus on the interaction between the modes of the cavity and the membrane with the pre-stress. Here, in order to consider several modes of the acoustic cavity, we use the marks $P_{i}$ and $p_{i}$ to respectively represent each mode shape and its corresponding acoustic pressure (i=1,2,3,…,N). Thus, the acoustic pressure inside the acoustic cavity is represented in the following form:

(2) $$p_{r}\left(x,y,z,t\right)=\overset{N}{\underset{i=1}{\sum }}\left(P_{i}\left(x,y,z\right)p_{i}\left(t\right)\right)$$

For the membrane, in this paper, one DOF model based on the membrane in Reference [17] is taken into account. In References [21,22,23], the membrane was mounted on one wall of the cavity and analyzed for the TET without the pre-stress. Here, the influence of the pre-stress of the membrane for the TET is analyzed, where the term of linear stiffness is used to present the pre-stress. We assume if the size of the membrane on the wall is small by comparing to the size of the acoustic cavity and the several low frequency modes are considered, the acoustic pressure in contact with the membrane is uniform and defined to equal to the value at the center of the membrane. Thus, the equation of the membrane is as follows:

(3) $$m_{m}\ddot{q}+k_{1}{\left(\frac{f_{1}}{f_{0}}\right)}^{2}q+k_{1}\eta \dot{q}+k_{3}\left(q^{3}+2\eta q^{2}\dot{q}\right)=\frac{s_{m}}{2}\overset{N}{\underset{i=1}{\sum }}\left(P_{i}\left(x_{m},y_{m},z_{m}\right)p_{i}\left(t\right)\right),$$

where,

(4) $$m_{m}=\frac{\rho_{m}hs_{m}}{3},\;k_{1}=\frac{1.015^{4}\pi^{5}}{36}\frac{Eh^{3}}{\left(1-\upsilon^{2}\right)R^{2}},\;f_{0}=\frac{1}{2\pi }\sqrt{\frac{1.015^{4}\pi^{4}}{12}\frac{Eh^{2}}{\left(1-\upsilon^{2}\right)\rho_{m}R^{4}}},\;k_{3}=\frac{8\pi Eh}{3\left(1-\upsilon^{2}\right)R^{2}},\;s_{m}=\pi R^{2}$$

q(t) is the transversal displacement of the membrane center (direction $O_{x}$ in Figure 1) and $p_{i}\left(t\right)$ is the acoustic pressure amplitude inside the cavity. The coefficients $k_{1}$ and $k_{3}$ are the linear and nonlinear stiffness, respectively. $f_{1}$ and $f_{0}$ are, respectively, the first resonance frequency of the membrane with and without the pre-stress. R, h, E, ν, η, and $\rho_{m}$ are the radius, the thickness, Young’s modulus, Poisson’s ratio, the viscous parameter, and the density of the membrane, respectively.

### 2.2. Coupling Between Several Acoustic Modes and a Nonlinear Membrane

The membrane is mounted on the wall of the cavity by the clamped boundary condition. Thus, the equations that govern the acoustic pressure inside the acoustic cavity are as follows:

(5) $$\frac{1}{c_{0}^{2}}\frac{\partial^{2}p_{r}}{\partial t^{2}}-\Delta p_{r}=f\left(x_{f},y_{f},z_{f},t\right)\;\mathrm{in}\;\Omega ,\;\;\frac{\partial p_{r}}{\partial n}=0\;\;\;\mathrm{on}\;\partial \Omega_{1},\;\rho_{a}\frac{\partial^{2}w}{\partial t^{2}}=-\frac{\partial p_{r}}{\partial n}\;\;\;\mathrm{on}\;\partial \Omega_{m}\;\;\left(\partial \Omega =\partial \Omega_{1}+\partial \Omega_{m}\right),$$

where $f\left(x_{f},y_{f},z_{f},t\right)$ is the source of force inside the cavity and $\left(x_{f},y_{f},z_{f}\right)$ is the position of the source. Ω, ∂Ω and $\partial \Omega_{m}$ are the internal volume of the cavity, the surface of the cavity and the surface of the membrane, respectively. $\partial \Omega_{1}$ is the surface of the cavity without the surface of the membrane. $\rho_{a}$ and $c_{0}$ are the density of the air and the sound velocity, respectively.

The system is performed a Rayleigh–Ritz reduction by using the mode shape $P_{i}$ as a single shape function for $p_{r}$ and for the test function $\delta p_{r}\left(x,y,z,t\right)=\sum_{i=1}^{N}P_{i}\left(x,y,z\right)\delta p_{i}\left(t\right)$ [22,23]:

(6) $$\int_{\Omega }\left(\frac{1}{c_{0}^{2}}\frac{\partial^{2}p_{r}}{\partial t^{2}}\delta p_{r}-\Delta p_{r}\delta p_{r}\right)d\Omega =\int_{\Omega }\left(f\left(x_{f},y_{f},z_{f},t\right)\delta p_{r}\right)d\Omega \;\Rightarrow \int_{\Omega }\frac{1}{c_{0}^{2}}\frac{\partial^{2}p_{r}}{\partial t^{2}}\delta p_{r}d\Omega +\int_{\Omega }\vec{\mathrm{grad}}p_{r}\cdot \vec{\mathrm{grad}}\delta p_{r}d\Omega -\int_{\partial \Omega_{m}}\frac{\partial p_{r}}{\partial n}\delta p_{r}d\Omega =\int_{\Omega }f\left(x_{f},y_{f},z_{f},t\right)\delta p_{r}d\Omega$$

Then, the following equations are obtained:

(7) $$\begin{array}{l} m_{a1}{\ddot{p}}_{1}+k_{a1}p_{1}+\frac{\rho_{a}^{2}c_{0}^{2}s_{m}}{2}P_{1}\left(x_{m},y_{m},z_{m}\right)\ddot{q}=\varepsilon_{1}f\left(t\right),\\ \ldots ,\\ m_{ai}{\ddot{p}}_{i}+k_{ai}p_{i}+\frac{\rho_{a}^{2}c_{0}^{2}s_{m}}{2}P_{i}\left(x_{m},y_{m},z_{m}\right)\ddot{q}=\varepsilon_{i}f\left(t\right),\\ \ldots ,\\ m_{aN}{\ddot{p}}_{N}+k_{aN}p_{N}+\frac{\rho_{a}^{2}c_{0}^{2}s_{m}}{2}P_{N}\left(x_{m},y_{m},z_{m}\right)\ddot{q}=\varepsilon_{N}f\left(t\right), \end{array}$$

where,

(8) $$V=L_{x}L_{y}L_{z},\;m_{ai}=\frac{\rho_{a}V}{\delta_{i}},\;k_{ai}=\frac{\rho_{a}V\omega_{i}^{2}}{\delta_{i}},\;\varepsilon_{i}=\rho_{a}c_{0}^{2}P_{i}\left(x_{f},y_{f},z_{f}\right)$$

$\delta_{i}$ and $\omega_{i}$ equal to $\left(2-\delta_{l0}\right)\left(2-\delta_{m0}\right)\left(2-\delta_{n0}\right)$ and $\pi c_{0}\sqrt{{\left(l/L_{x}\right)}^{2}+{\left(m/L_{y}\right)}^{2}+{\left(n/L_{z}\right)}^{2}}$, respectively. 

For $\delta_{l0},\;\delta_{m0},\;\delta_{n0}$,

(9) $$l=0,\;\delta_{l0}=1;\;l\neq 0,\;\delta_{l0}=0;\;\;m=0,\;\delta_{m0}=1;\;m\neq 0,\;\delta_{m0}=0;\;\;n=0,\;\delta_{n0}=1;\;n\neq 0,\;\delta_{n0}=0;$$

Finally, the general model of the system with several acoustic modes of 3D acoustic cavity and one nonlinear membrane is obtained by dividing the corresponding mass of each equation of Equations (3) and (7), replacing the pressure amplitude by the displacement amplitude $u_{i}$ $\left(p_{i}\left(t\right)=\rho_{a}c_{0}\omega_{i}u_{i}\left(t\right)\right)$ and introducing a coefficient $\lambda_{i}$ for the acoustic damping:

(10) $$\begin{array}{l} {\;\ddot{u}}_{1}+\mu_{1}{\dot{u}}_{1}+\omega_{1}^{2}u_{1}+\phi_{1}\ddot{q}=\alpha_{1}f\left(t\right),\\ \;\ldots ,\\ \;{\ddot{u}}_{i}+\mu_{i}{\dot{u}}_{i}+\omega_{i}^{2}u_{i}+\phi_{i}\ddot{q}=\alpha_{i}f\left(t\right),\\ \;\ldots ,\\ \;{\ddot{u}}_{N}+\mu_{N}{\dot{u}}_{N}+\omega_{N}^{2}u_{N}+\phi_{N}\ddot{q}=\alpha_{N}f\left(t\right),\\ \ddot{q}+\frac{k_{1}}{m_{m}}{\left(\frac{f_{1}}{f_{0}}\right)}^{2}q+\mu_{m1}\dot{q}+\mu_{m2}q^{2}\dot{q}+\beta q^{3}-\overset{N}{\underset{i=1}{\sum }}\gamma_{i}u_{i}=0, \end{array}$$

where,

(11) $$\mu_{i}=\frac{\lambda_{i}}{m_{ai}},\;\phi_{i}=\frac{s_{m}\rho_{a}c_{0}}{2m_{ai}\omega_{i}}P_{i}\left(x_{m},y_{m},z_{m}\right),\;\mu_{m1}=\frac{k_{1}\eta }{m_{m}},\;\mu_{m2}=\frac{2k_{3}\eta }{m_{m}},\;\beta =\frac{k_{3}}{m_{m}},\;\alpha_{i}=\frac{\varepsilon_{i}}{m_{ai}\rho_{a}c_{0}\omega_{i}},\;\gamma_{i}=\frac{s_{m}\rho_{a}c_{0}\omega_{i}}{2m_{m}}P_{i}\left(x_{m},y_{m},z_{m}\right)$$

For the parameters, we choose the dimensions of the cavity are $L_{x}=1$ m, $L_{y}=2.2$ m, $L_{z}=1.6$ m. The position of the membrane is $x_{m}=L_{x},\;y_{m}=L_{y}/6,\;z_{m}=L_{z}/6$ and the position of the source of forcing is $x_{f}=L_{x}/3,\;y_{f}=L_{y}/3,\;z_{f}=L_{z}/3$. The values of the membrane and air parameters are: R=0.04 m, h=0.00039 m, η=0.000062 s^−1^, E=1.48 MPa, υ=0.49, $\rho_{m}=980$ kg m^−3^, $\rho_{a}=1.3$ kg m^−3^, and $c_{0}=350$ m s^−1^. And they are fixed along the paper. The units of p(t), f(t), Frequency and time are Pa, m/s^2^, Hz and s, respectively and the unit of the amplitude of u(t) and q(t) is m.

## 3. Influence of the Pre-Stress of the Membrane for the TET

In Reference [21], the TET phenomenon of the system with one mode of the acoustic cavity and one membrane without the pre-stress was observed according to the strongly modulated response (SMR) [25,26]. And the desired working zone for the membrane NES as the forcing level interval was defined based on the first destabilization of the resonance peak and the appearance of an additional branch of periodic regimes. In order to analyze the influence of the pre-stress of the membrane for the TET, a system comprised by the first mode of the acoustic cavity and one membrane with the pre-stress is considered in the following form:

(12) $$\ddot{u}+\mu \dot{u}+\omega_{010}^{2}u+\phi \ddot{q}=F\left(t\right),\;\ddot{q}+\omega_{m}^{2}q+\mu_{m1}\dot{q}+\mu_{m2}q^{2}\dot{q}+\beta q^{3}-\gamma u=0,$$

where,

(13) $$\omega_{010}=\frac{\pi c_{0}}{L_{y}},\;\;F\left(t\right)=\frac{\varepsilon_{1}f\left(t\right)}{m_{a}\rho_{a}c_{0}\omega_{010}},\;\;\omega_{m}=\sqrt{\frac{k_{1}}{m_{m}}{\left(\frac{f_{1}}{f_{0}}\right)}^{2}}$$

**Figure 2:**
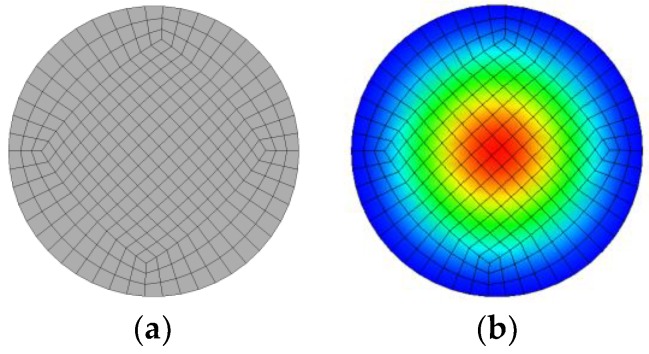
The membrane. (**a**): the finite element model and (**b**): the first mode with $f_{0}=5.08\;Hz$.

Here, $\omega_{m}$ represents the term of the pre-stress of the membrane. For different pre-stress, the different $f_{1}$ values are taken into account based on the test results in Reference [17]. However, $f_{0}$ is a constant for the membrane without the pre-stress. In this paper, the FEA method is used to calculate the value of $f_{0}$ to validate the formulae of $f_{0}$. The FE model of the membrane is built and the first mode of the membrane is obtained, as shown in Figure 2. We can see that the values obtained by FEA and the theoretical formulae are same as $f_{0}=5.08\;Hz$.

The term of $\omega_{m}^{2}q$ is just added in the system (12) with the pre-stress of the membrane compared with the system without the pre-stress of the membrane in Reference [21]. Here, by using the harmonic balance method (HBM) with a single harmonic term, the periodic forced responses of the system are investigated. In Equation (12), the nonlinear terms are $q^{2}\dot{q}$ and $q^{3}$. Thus, in order to get the easy calculation, we set $\dot{q}\left(0\right)=0$ and take the force F(t) and the displacements u(t) and q(t) as follows:

(14) $$F\left(t\right)=F_{1c}cos\left(\omega t\right)+F_{1s}sin\left(\omega t\right),\;u\left(t\right)=u_{1c}cos\left(\omega t\right)+u_{1s}sin\left(\omega t\right),\;q\left(t\right)=q_{1c}cos\left(\omega t\right)$$

By substituting the solutions in Equation (14) into Equation (12) and neglecting the higher harmonic in 3ω, the following algebraic equations are obtained:

(15) $$\left(\omega_{010}^{2}-\omega^{2}\right)u_{1c}+\mu \omega u_{1s}-\phi \omega^{2}q_{1c}=F_{1c},\;\left(\omega_{010}^{2}-\omega^{2}\right)u_{1s}-\mu \omega u_{1c}=F_{1s},\;\frac{3}{4}\beta q_{1c}^{3}+\omega_{m}^{2}q_{1c}-\omega^{2}q_{1c}-\gamma u_{1c}=0,\;-\frac{1}{4}\mu_{m2}\omega q_{1c}^{3}-\mu_{m1}\omega q_{1c}-\gamma u_{1s}=0,\;F_{1}=F_{1c}+F_{1s},\;u_{1}=u_{1c}+u_{1s}$$

**Figure 3:**
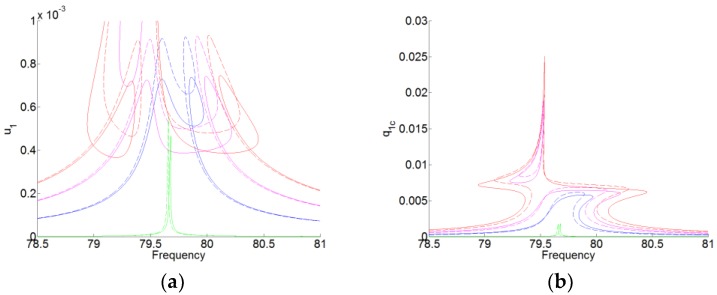
The periodic forced responses of the system for $F_{1}=\left[0.01:0.6:2\right]$. (**a**) u(t) and (**b**) q(t). The dashed lines represent the responses without the pre-stress of the membrane. The solid lines represent the responses with the pre-stress of the membrane ($f_{1}=30\;Hz$).

For Equation (15), there are eight unknown parameters. We take the amplitude of the membrane $q_{1c}$ and the angular frequency of the force ω as the master parameters. Thus, the response surfaces are obtained with the pre-stress of the membrane ($f_{1}=30\;Hz$), as shown in Figure 3. In Figure 3, the dashed lines represent the responses without the pre-stress of the membrane, which are the same results in Reference [21]. The solid lines represent the responses with the pre-stress of the membrane. We can see that according to the form of the responses, the TET phenomenon can occur for the system with the membrane with the pre-stress. And the membrane with the pre-stress can reduce the amplitude of the acoustic displacement, which is the plateau of the amplitude of $u_{1}$.

In Reference [21], the desired working zone for the membrane NES without the pre-stress as the forcing level interval was defined based on the first destabilization of the resonance peak and the appearance of an additional branch of periodic regimes. The analytical formula of the two thresholds $F_{b}$ and $F_{e}$ are defined. In order to analyze the influence of the pre-stress of the membrane for the TET of the system, three configurations for the pre-stress of the membrane are chosen, which are $f_{1}=30\;Hz$, $f_{1}=50\;Hz$ and $f_{1}=65\;Hz$, respectively.

**Figure 4:**
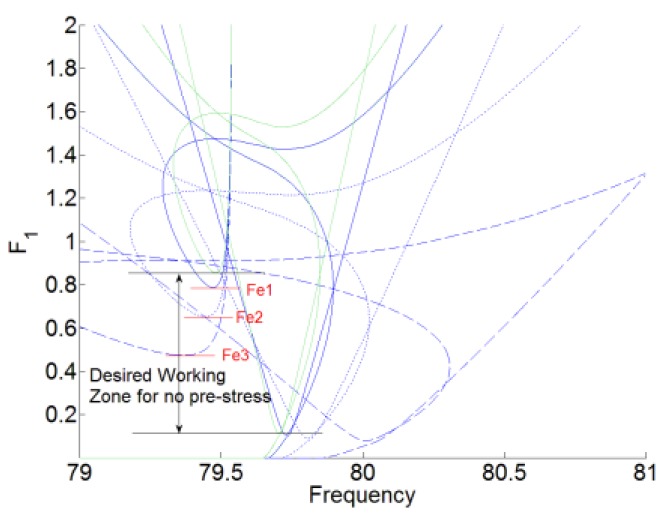
The desired working zone for the membrane nonlinear energy sink (NES) with three different pre-stress. The green and solid lines represent no the pre-stress. The blue and solid lines are for $f_{1}=30\;Hz$. The blue and dotted lines are for $f_{1}=50\;Hz$. The blue and dashed lines are for $f_{1}=65\;Hz$.

**Figure 5:**
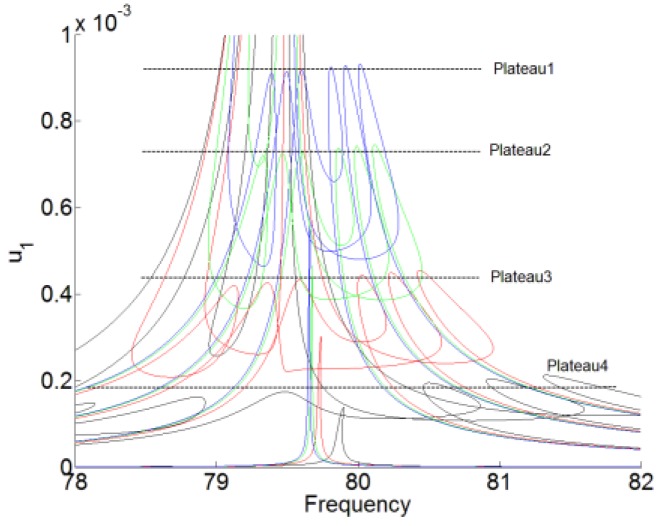
The plateau of the amplitude $u_{1}$ for the acoustic displacement. The forced response is under the forcing levels $F_{1}=\left[0.01:0.6:2\right]$. The blue lines represent no the pre-stress, marked by ‘Plateau1’. The green lines are for $f_{1}=30\;Hz$, marked by ‘Plateau2’. The red lines are for $f_{1}=50\;Hz$, marked by ‘Plateau3’. The black lines are for $f_{1}=65\;Hz$, marked by ‘Plateau4’.

Figure 4 and Figure 5 show the desired working zone for the membrane NES and the plateau of the amplitude $u_{1}$ for the acoustic displacement with three different pre-stress, which are represented by $f_{1}=30\;Hz$, $f_{1}=50\;Hz$, and $f_{1}=65\;Hz$. $F_{e1}$, $F_{e2}$, and $F_{e3}$ represent the ending thresholds for $f_{1}=30\;Hz$, $f_{1}=50\;Hz$, and $f_{1}=65\;Hz$, respectively. Plateau1, Plateau2, Plateau3, and Plateau4 represent the plateau of the amplitude of for the acoustic displacement for no pre-stress, $f_{1}=30\;Hz$, $f_{1}=50\;Hz$, and $f_{1}=65\;Hz$, respectively. By comparing with no pre-stress of the membrane, which is represented by the green and solid lines, the higher of the first resonance frequency $f_{1}$, the narrower desired working zone for the membrane NES. And the beginning threshold $F_{b}$ is nearly unchanged, while the ending threshold $F_{e}$ decreases with the increase of the resonance frequency $f_{1}$. But, with the higher pre-stress, we also can obtain the lower value for the plateau of the amplitude of $u_{1}$ (the value of the response suppression) and the larger bandwidth of frequency for the noise suppression. 

**Figure 6:**
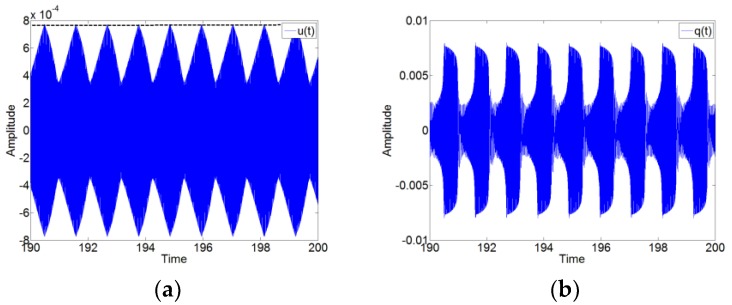
The time series for the displacement u(t) and q(t) with the forcing level $F_{1}=1.2$ and the excitation frequency 79.9 Hz. (**a**) u(t) and (**b**) q(t). The dashed and black line represents the plateau of the amplitude of $u_{1}$.

The numerical simulations of the system (12) are performed by using the 4th and 5th order Runge–Kutta method to validate the TET phenomenon of the system with the pre-stress of the membrane. Here, we only take an example $f_{1}=30\;Hz$ to analyze the numerical results. Figure 6 shows the time series for the displacements u(t) and q(t) with the forcing level $F_{1}=1.2$ and the excitation frequency 79.9 Hz. We can see that the responses of the system show the SMR. Thus, the TET phenomenon occurs for the system with the pre-stress of the membrane. Meanwhile, the value of the plateau for $f_{1}=30\;Hz$ in Figure 6a by the dashed and black line show a good correspondence with the value of the plateau in Figure 5 by ‘Plateau2’.

## 4. Two Modes with One Membrane

Before analyzing the TET phenomenon of the system with several modes of the acoustic cavity and one membrane, a system with two modes of the acoustic cavity and one membrane is firstly studied to find the relation between different modes of the cavity and the membrane and whether the membrane can work for different modes. Based on Equation (7), the system with two modes $P_{1}=P_{010}$ and $P_{2}=P_{001}$ of the acoustic cavity (and also the static mode $P_{0}=P_{000}$) and one membrane can be represented without considering the influence of pre-stress of the membrane. Here, because of the static mode $P_{0}=P_{000}$, the pressure amplitude could not be replaced by the displacement amplitude $\left(p_{i}\left(t\right)=\rho_{a}c_{0}\omega_{i}u_{i}\left(t\right)\right)$. Thus, the system reads in the following form:

(16) $${\ddot{p}}_{0}+\lambda_{0}{\dot{p}}_{0}+\phi_{0}^{*}\ddot{q}=\alpha_{0}^{*}f\left(t\right),\;{\ddot{p}}_{1}+\lambda_{1}{\dot{p}}_{1}+\omega_{1}^{2}p_{1}+\phi_{1}^{*}\ddot{q}=\alpha_{1}^{*}f\left(t\right),\;{\ddot{p}}_{2}+\lambda_{2}{\dot{p}}_{2}+\omega_{2}^{2}p_{2}+\phi_{2}^{*}\ddot{q}=\alpha_{2}^{*}f\left(t\right),\;\ddot{q}+\mu_{m1}\dot{q}+\mu_{m2}q^{2}\dot{q}+\beta q^{3}-\gamma_{0}^{*}p_{0}-\gamma_{1}^{*}p_{1}-\gamma_{2}^{*}p_{2}=0$$

where,

(17) $$\lambda_{i}=\mu_{i}m_{ai},\;\phi_{i}^{*}=\frac{s_{m}\rho_{a}^{2}c_{0}^{2}}{2m_{ai}}P_{i}\left(x_{m},y_{m},z_{m}\right),\;\alpha_{i}^{*}=\frac{\varepsilon_{i}}{m_{ai}},\;\;\omega_{1}=\frac{\pi c_{0}}{L_{y}},\;\omega_{2}=\frac{\pi c_{0}}{L_{z}},\;\gamma_{i}^{*}=\frac{s_{m}}{2m_{m}}P_{i}\left(x_{m},y_{m},z_{m}\right)$$

### 4.1. Nonlinear Normal Modes

In order to analyze the NNMs of the system (16), we remove the force and all the damping in the system, the system becomes:

(18) $${\ddot{p}}_{0}+\phi_{0}^{*}\ddot{q}=0,\;{\ddot{p}}_{1}+\omega_{1}^{2}p_{1}+\phi_{1}^{*}\ddot{q}=0,\;{\ddot{p}}_{2}+\omega_{2}^{2}p_{2}+\phi_{2}^{*}\ddot{q}=0,\;\ddot{q}+\beta q^{3}-\gamma_{0}^{*}p_{0}-\gamma_{1}^{*}p_{1}-\gamma_{2}^{*}p_{2}=0$$

By using HBM with a single term, the motion of the system (18) are as follows:

(19) $$p_{0}\left(t\right)=p_{0c}cos\left(\omega t\right),\;p_{1}\left(t\right)=p_{1c}cos\left(\omega t\right),\;p_{2}\left(t\right)=p_{2c}cos\left(\omega t\right),\;q\left(t\right)=q_{1c}cos\left(\omega t\right)$$

Then, Equation (19) is introduced into the system (18) and the following three algebraic equations are obtained by neglecting the higher harmonics in 3ω and expressing $p_{0c}=-\phi_{0}^{*}q_{1c}$:

(20) $$\left(\omega_{1}^{2}-\omega^{2}\right)p_{1c}-\phi_{1}^{*}\omega^{2}q_{1c}=0,\;\left(\omega_{2}^{2}-\omega^{2}\right)p_{2c}-\phi_{2}^{*}\omega^{2}q_{1c}=0,\;\phi_{0}^{*}\gamma_{0}^{*}q_{1c}-\gamma_{1}^{*}p_{1c}-\gamma_{2}^{*}p_{2c}-\omega^{2}q_{1c}+\frac{3}{4}\beta q_{1c}^{3}=0$$

**Figure 7:**
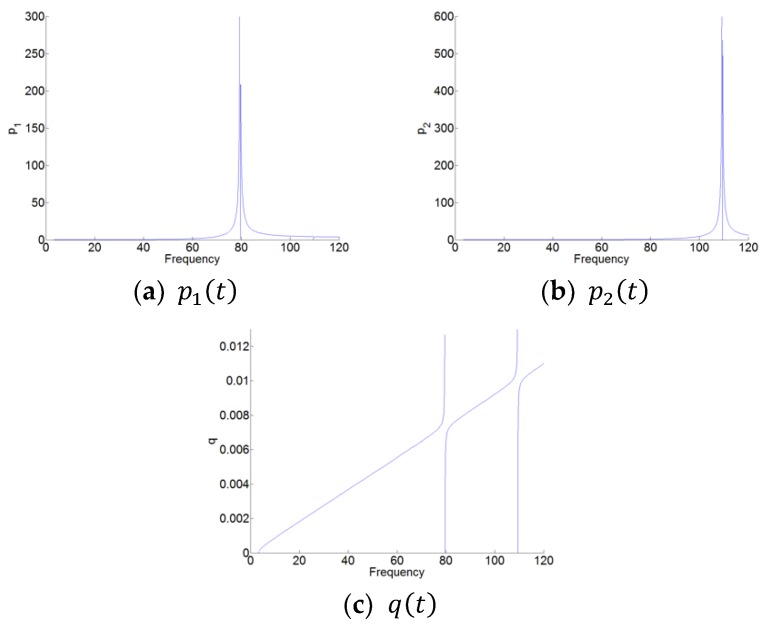
The nonlinear normal modes (NNMs) of the system-amplitudes. (**a**) $p_{1}\left(t\right)$, (**b**) $p_{2}\left(t\right)$, and (**c**) q(t).

**Figure 8:**
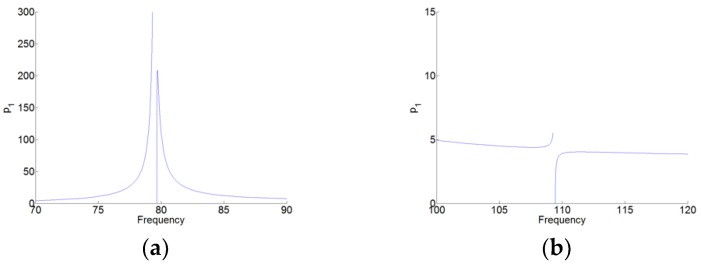
Zoom of $p_{1}\left(t\right)$ in Figure 7a. (**a**,**b**) around the two resonant frequencies of the cavity.

**Figure 9:**
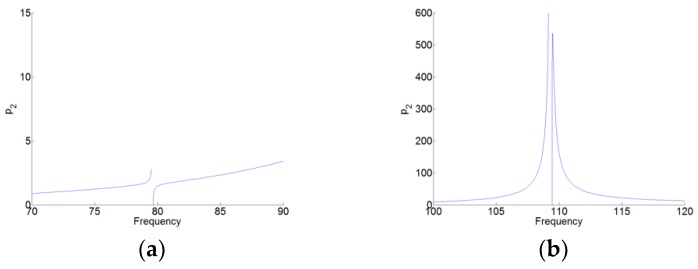
Zoom of $p_{2}\left(t\right)$ in Figure 7b. (**a**,**b**) around the two resonant frequencies of the cavity.

Here, the absolute values of the amplitude $p_{1c}$, $p_{2c}$ and $q_{1c}$ are represented, as shown in Figure 7. Figure 8 and Figure 9 are the zoom of $p_{1}\left(t\right)$ and $p_{2}\left(t\right)$, respectively. We can see that in Figure 8, the amplitude of $p_{1}\left(t\right)$ around the first resonant of the cavity (here, it’s the natural angular frequency $\omega_{1}$ of the cavity) is much higher than that around the second resonant frequency of the cavity (here, it is the natural angular frequency $\omega_{2}$ of the cavity). While in Figure 9, the amplitude of $p_{2}\left(t\right)$ around the angular frequency $\omega_{2}$ is much higher than that around the angular frequency $\omega_{1}$. Therefore, for $p_{1}\left(t\right)$ and $p_{2}\left(t\right)$, there are the high amplitudes around only one resonant frequency and around its natural angular frequency $\omega_{1}$ and $\omega_{2}$, respectively. Meanwhile, there is the weak coupling between the oscillators $p_{1}\left(t\right)$ and $p_{2}\left(t\right)$. For q(t) in Figure 7c, we can see observe that q(t) has the large amplitudes around the two natural angular frequencies.

### 4.2. Forced Responses

**Figure 10:**
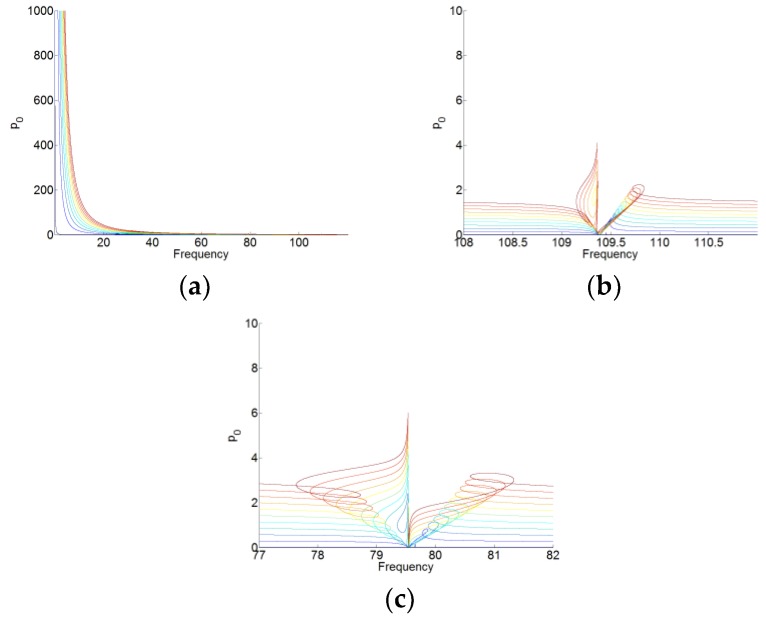
(**a**) Responses of $p_{0}\left(t\right)$ for $F_{1}=\left[0.01:2:20.01\right]$. (**b**,**c**) are the zooms of (**a**) around two resonant frequencies of the cavity.

**Figure 11:**
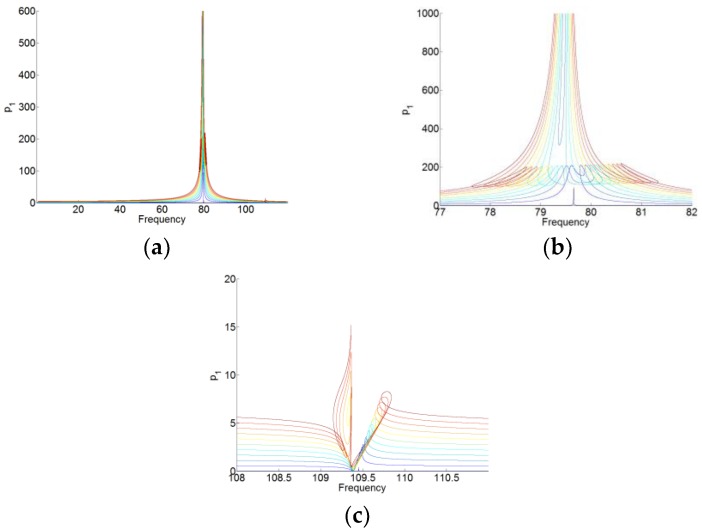
(**a**) Responses of $p_{1}\left(t\right)$ for $F_{1}=\left[0.01:2:20.01\right]$. (**b**,**c**) are the zooms of (**a**) around two resonant frequencies of the cavity.

**Figure 12:**
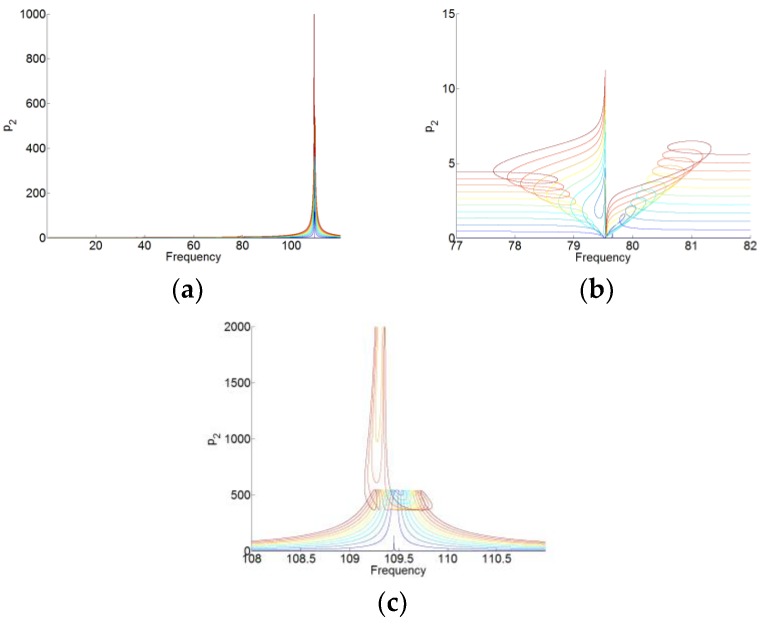
(**a**) Responses of $p_{2}\left(t\right)$ for $F_{1}=\left[0.01:2:20.01\right]$. (**b**,**c**) are the zooms of (**a**) around two resonant frequencies of the cavity.

**Figure 13:**
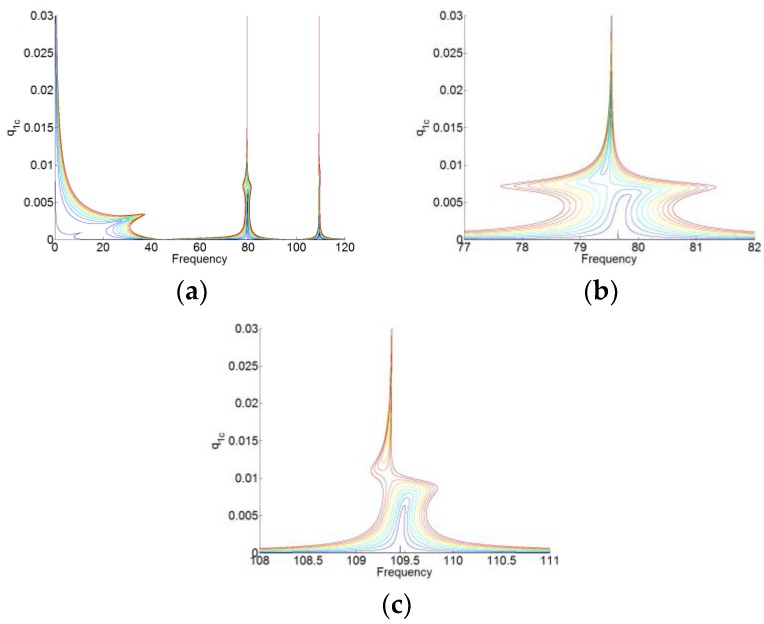
(**a**) Responses of q(t) for $F_{1}=\left[0.01:2:20.01\right]$. (**b**,**c**) are the zooms of (**a**) around two resonant frequencies of the cavity.

The solutions to periodic forcing of the system (16) are analyzed by using the same setting and method with the system (12), which are shown in Figure 10, Figure 11, Figure 12 and Figure 13. Here, we set $f\left(t\right)=F_{1c}cos\left(\omega t\right)+F_{1s}sin\left(\omega t\right)$ and $F_{1}=F_{1c}+F_{1s}$. The gradual color stands for the level of forcing $F_{1}$, where the blue curve indicates a low level of forcing $F_{1}$ and the red one indicates a high level of forcing $F_{1}$.

For $p_{0}\left(t\right)$, $p_{1}\left(t\right)$ and $p_{2}\left(t\right)$, we can see that when the membrane works in the desired zone for the TET phenomenon, and they vibrate only around its natural angular frequency $0,\;\omega_{1}$ and $\omega_{2}$, respectively. Around its natural angular frequency, the amplitude of the responses is also much higher than that around the other resonant frequency. Therefore, there is also weak coupling between the responses of $p_{0}\left(t\right)$, $p_{1}\left(t\right)$, and $p_{2}\left(t\right)$. In Figure 13, we can see that there are always the large amplitudes of the responses around the resonant frequencies for q(t). The membrane can work for two resonant angular frequency $\omega_{1}$ and $\omega_{2}$ of the cavity. Because of the weak coupling between the NNMs and the forced responses of the system (16), it is useful to analyze the NNMs and the forced responses of the system with each mode ($P_{1}=P_{010}$ and $P_{2}=P_{001}$ in the system of (16)) and the membrane.

**Figure 14:**
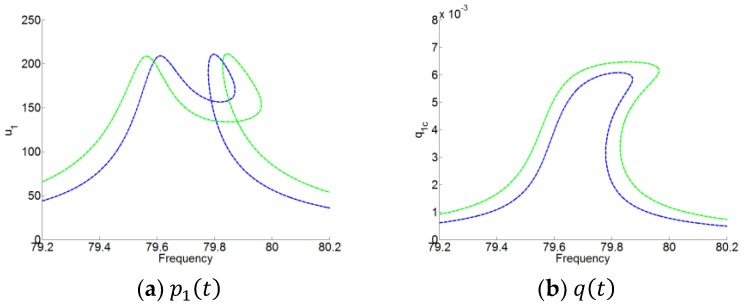
Responses of $p_{1}\left(t\right)$ and q(t) of the system for $F_{1}=2.01$ in blue curves and $F_{1}=3.01$ in green curves. Dashed curves stand for the responses of the system with the two modes $P_{010}$ and $P_{001}$ coupled by the membrane. Solid curves stand for the responses of the system only with the mode $P_{010}$ coupled by the membrane. (**a**) $p_{1}\left(t\right)$ and (**b**) q(t).

The forced responses of the system (16) are compared with that of the system with each mode ($P_{1}=P_{010}$) and the membrane, as shown in Figure 14. We can observe that around the resonant frequency (for the system with the mode $P_{010}$ in $\omega_{1}$), the responses of the system with one mode are the same as those of the system with the two modes. Therefore, for looking for the forced responses of the system (16), we can firstly separate it to two new systems composed of each mode of the cavity coupled by the membrane. Then, the responses of the two new systems with two DOFs could be easily analyzed.

### 4.3. Numerical Simulations

**Figure 15:**
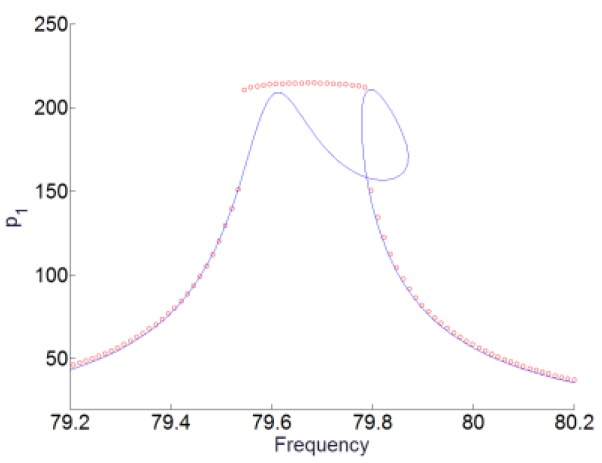
Numerical and analytical responses of $p_{1}\left(t\right)$ for the level of forcing $F_{1}=2.01$. Red curves with circle stand for numerical results. Blue curves stand for analytical results.

**Figure 16:**
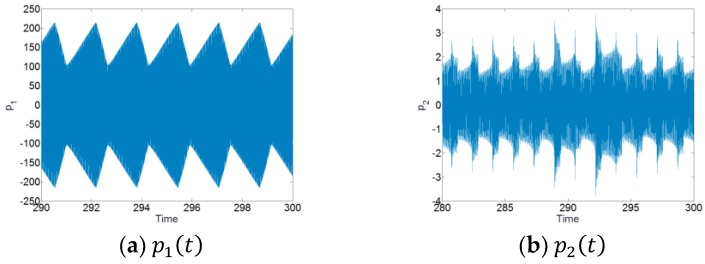

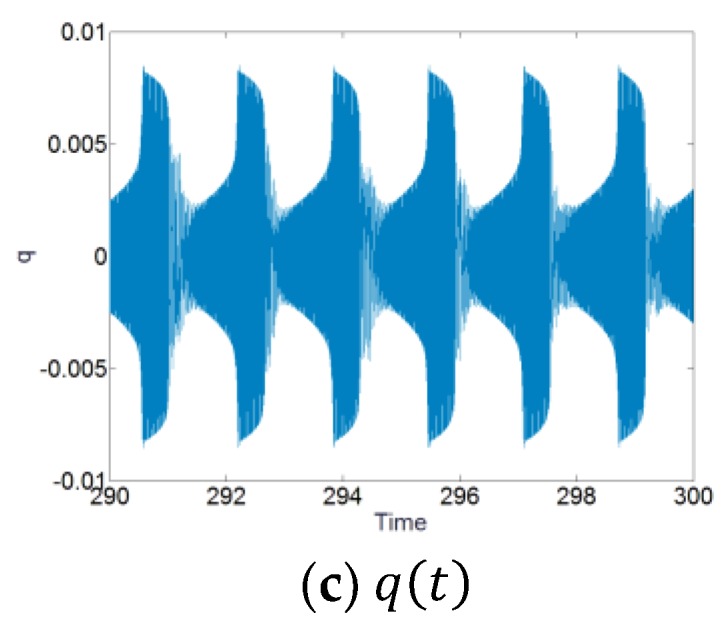
The time series for the system with the level of forcing $F_{1}=2.01$ and the frequency of excitation 79.7 Hz. (**a**) $p_{1}\left(t\right)$, (**b**) $p_{2}\left(t\right)$, and (**c**) q(t).

The numerical simulations of the system (16) are also performed by using the 4th and 5th order Runge–Kutta method to study the TET phenomenon of the system and also to validate the analytical results. We will here choose the level of forcing $F_{1}=2.01$. Based on the range of frequency of the TET phenomenon, the frequency of excitation is chosen for the range [79.2 Hz, 80.2 Hz]. The maximal amplitudes of $p_{1}\left(t\right)$ obtained by numerical integration around the first resonant angular frequency $\omega_{1}$ are plotted together with the analytical responses of the system shown in Figure 15. The numerical results are marked with red circles. We can see that the numerical responses of $p_{1}\left(t\right)$ show a good agreement with the analytical results. In Figure 16, the time series of the system for the forcing $F_{1}=2.01$ and the frequency of excitation 79.7 Hz is shown to investigate the responses. We can see that for $p_{1}\left(t\right)$ and q(t), there are the SMR and the TET phenomenon of the system occurs. And for $p_{2}\left(t\right)$, the amplitudes of the responses are much smaller than that of $p_{1}\left(t\right)$. Therefore, the numerical simulations are in line with the analytical predictions.

## 5. Several Modes with One Membrane

### 5.1. The Modes of 3D Acoustic Cavity

The natural frequencies between the frequency range 20–200 Hz of the cavity are represented according to the frequency equation $f_{lmn}=\frac{c_{0}}{2}\sqrt{{\left(l/L_{x}\right)}^{2}+{\left(m/L_{y}\right)}^{2}+{\left(n/L_{z}\right)}^{2}}$, as follows in Table 1. 

Finally, in order to control the low frequency broadband noise (20–200 Hz) inside the cavity, the system comprised by seven DOFs linear oscillators and the membrane is built based on Equation (10).

### 5.2. The Thresholds of the Desired Working Zone

For the acoustic damping of the modes, we set all damping coefficients $\mu_{i}=0.014$, which are weak for the modes of the cavity. The periodic forced responses of the system with seven modes coupled by the membrane can be obtained by analyzing the system with each mode coupled by the membrane. For a two DOFs system with one acoustic mode and the membrane, the forced responses of the system can be easily obtained by using the HBM with one term.

According to the results in Reference [21], we studied the extrema of the curves for the responses u(t) versus ω under the level of forcing $F_{1}$. The desired working zone of the membrane was determined by analyzing the zone with three extrema. Then, by using the HBM method, the analytical expression of the threshold $F_{b}$ for the beginning of the zone was obtained:

(21) $$F_{b}=\bar{F_{1}}=\sqrt{{\left(\lambda \bar{\omega }{\bar{p}}_{1s}\right)}^{2}+{\left(\left(\omega_{010}^{2}-{\bar{\omega }}^{2}\right){\bar{p}}_{1s}-\lambda \bar{\omega }{\bar{p}}_{1c}\right)}^{2}}$$

And the analytical expression for the value of the plateau is also obtained:

(22) $$\bar{p_{1}}=\frac{1}{\gamma }\sqrt{{\left(\frac{3}{4}\beta {\bar{q}}_{1c}{}^{3}-{\bar{\omega }}^{2}{\bar{q}}_{1c}\right)}^{2}+{\left(\frac{1}{4}\mu_{m2}\bar{\omega }{\bar{q}}_{1c}{}^{3}+\mu_{m2}\bar{\omega }{\bar{q}}_{1c}\right)}^{2}}$$

where, $\bar{\omega },\;{\bar{p}}_{1c},\;{\bar{p}}_{1s},{\bar{q}}_{1c}$ represent the solutions of the limit point.

About the threshold $F_{e}$ for the ending of the zone, a formula of the level of forcing $F_{1}$ depending only on ω ($\omega <\omega_{010}$ for the mode $P_{010}$) is obtained:

(23) $$q_{1c}=\sqrt{\frac{4}{3\beta }\left(\omega^{2}-\frac{\gamma \phi \omega^{2}}{\omega^{2}-\omega_{010}^{2}}\right)},\;p_{1c}=\frac{1}{\gamma }\left(\frac{3}{4}\beta q_{1c}^{3}-\omega^{2}q_{1c}\right),\;\;p_{1s}=-\frac{1}{\gamma }\left(\frac{1}{4}\mu_{m2}\omega q_{1c}^{3}+\mu_{m1}\omega q_{1c}\right),\;\;F_{1}=\sqrt{{\left(\left(\omega_{010}^{2}-\omega^{2}\right)p_{1c}+\lambda \omega p_{1s}-\phi \omega^{2}q_{1c}\right)}^{2}+{\left(\left(\omega_{010}^{2}-\omega^{2}\right)p_{1s}-\lambda \omega p_{1c}\right)}^{2}}$$

**Figure 17:**
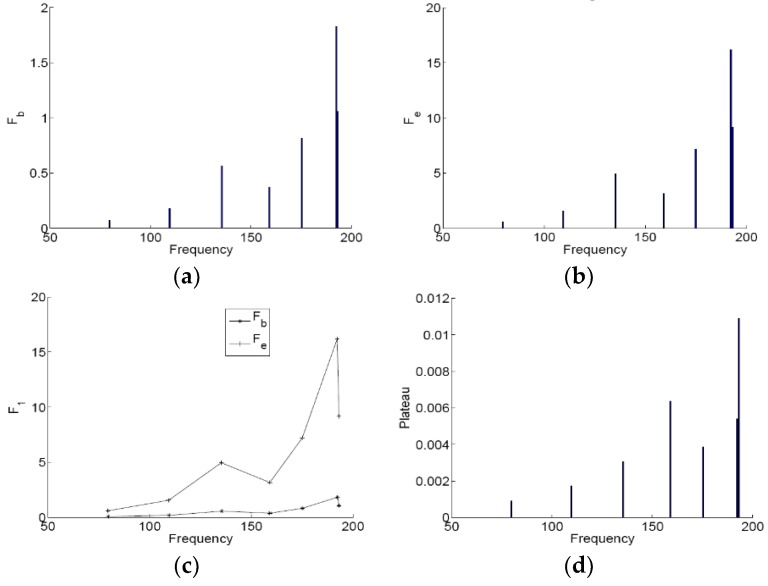
The two thresholds $F_{b}$ and $F_{e}$ and the plateau of the system composed of each mode coupled by the membrane. (**a**) the threshold $F_{b}$, (**b**) the threshold $F_{e}$, (**c**) the desired working zone and (**d**) the amplitude of the plateau.

Based on Equations (21)–(23), we can get the two thresholds $F_{b}$ and $F_{e}$ and the amplitude of the plateau of the system composed of each mode coupled by the membrane, as shown in Figure 17. We can observe that the system with each acoustic mode has different thresholds $F_{b}$ and $F_{e}$, namely that the membrane NES has the different desired working zone for the range of chosen frequencies. Therefore, the membrane could reduce the resonance peaks in the frequency range 20–200 Hz and control the low frequency broadband noise (20–200 Hz) inside 3D acoustic cavity. For a given 3D acoustic cavity, we can design the membrane by using the parametric results of the membrane to enhance the robustness and the effective TET range.

## 6. Discussion and Conclusions

In order to control the low frequency broadband noise (20–200 Hz) inside 3D acoustic cavity and consider the influence of the pre-stress for the TET, a general model of the system with several acoustic modes of 3D acoustic cavity and one nonlinear membrane is built. The analytical formula of the first resonance frequency for the membrane without the pre-stress is validated by the FEA method. For the results of the influence of the pre-stress of the membrane NES, if the membrane has the higher pre-stress (represented by the first resonance frequency with the pre-stress $f_{1}$), the narrower desired working zone for the membrane NES is obtained with no change for the beginning threshold $F_{b}$ and decrease for the ending threshold $F_{e}$. However, the lower value of the plateau of the amplitude of $u_{1}$ and the larger bandwidth of frequency for the noise suppression could be got. The results for the membrane with the pre-stress are validated by the numerical simulations.

A three DOFs system comprised by the membrane and two acoustic modes is then studied to demonstrate the membrane could work for the two acoustic modes. We can separate the two modes of the cavity to couple the membrane for looking for the solutions of the system according to the analytical results. Moreover, the numerical simulations are performed to validate the analytical predictions and the TET phenomenon. Finally, the multi-DOFs system with seven modes and the membrane is analyzed to get the desired working zone and the value of the plateau of the nonlinear membrane absorber for low frequency broadband noise. It will be helpful to design the nonlinear membrane NES according the dimension of a given 3D acoustic cavity to reduce the low frequency noise. It provides us a new treatment to control passively the low frequency broadband noise.

In this paper, for 3D acoustic cavity, the irregular acoustic cavity and the acoustic damping modes are not considered. For the nonlinear membrane, the membrane is not considered to be mounted on the flexible plate. Because these will cause a coupling between the acoustic cavity modes and also affect the forced responses inside 3D acoustic cavity. In future, different above conditions will be analyzed for the practical applications of the membrane. And the analytical and numerical results of the system will be also validated by the experimental methods. For other applications of the nonlinear membrane, the acoustic metamaterials based on the membrane will be taken in account and the sound energy harvesting based on TET by using the designed structures including the nonlinear membrane will be investigated. It may provide novel applications for the membrane and the secondary energy utilization of noise inside 3D acoustic cavity.

## Figures and Tables

**Table 1:** The natural frequencies between 20–200 Hz of the cavity.

l	m	n	Hz
0	1	0	79.8
0	0	1	109.38
0	1	1	135.28
0	2	0	159.08
1	0	0	175
1	1	0	192.15
0	2	1	193.03

## References

[1] Padhi T., Chandra M., Kar A., Kar A., Swamy M. (2017). Design and analysis of an improved hybrid active noise control system. Appl. Acoust..

[2] Hu L., Shi Y., Yang Q., Song G. (2016). Sound reduction at a target point inside an enclosed cavity using particle dampers. J. Sound Vib..

[3] Ihlenburg F. (1998). Finite Element Analysis of Acoustic Scattering.

[4] Wu T. (2000). Boundary Element Acoustics: Fundamentals and Computer Codes.

[5] Wu H., Ye W., Jiang W. (2015). Isogeometric finite element analysis of interior acoustic problems. Appl. Acoust..

[6] Fang N., Xi D., Xu J., Ambati M., Srituravanich W., Sun C., Zhang X. (2006). Ultrasonic metamaterials with negative modulus. Nat. Mater..

[7] Lee S., Park C., Seo Y., Wang Z., Kim C. (2009). Acoustic metamaterial with negative modulus. J. Phys. Condens. Matter.

[8] Cummer S., Christensen J., Alù A. (2016). Controlling sound with acoustic metamaterials. Nat. Rev. Mater..

[9] Gendelman O., Manevitch L., Vakakis A., M’Closkey R. (2001). Energy pumping in nonlinear mechanical oscillators: Part I—Dynamics of the underlying Hamiltonian systems. ASME J. Appl. Mech..

[10] Vakakis A., Gendelman O. (2001). Energy pumping in nonlinear mechanical oscillators: Part II—Resonance capture. ASME J. Appl. Mech..

[11] Gendelman O., Gourdon E., Lamarque C. (2006). Quasiperiodic energy pumping in coupled oscillators under periodic forcing. J. Sound Vib..

[12] Gourdon E., Alexander N., Taylor C., Lamarque C., Pernot S. (2007). Nonlinear energy pumping under transient forcing with strongly nonlinear coupling: Theoretical and experimental results. J. Sound Vib..

[13] Nucera F., Vakakis A., McFarland D., Bergman L., Kerschen G. (2007). Targeted energy transfers in vibro-impact oscillators for seismic mitigation. Nonlinear Dyn..

[14] Starosvetsky Y., Gendelman O. (2009). Vibration absorption in systems with a nonlinear energy sink: Nonlinear pumping. J. Sound Vib..

[15] Shao J., Wu X., Cochelin B. Targeted energy transfer in two degrees-of-freedom linear system coupled by one nonlinear absorber. Proceedings of the 21st International Congress on Sound and Vibration.

[16] Cochelin B., Herzog P., Mattei P.-O. (2006). Experimental evidence of energy pumping in acoustics. C. R. Mec..

[17] Bellet R., Cochelin B., Herzog P., Mattei P.-O. (2010). Experimental study of targeted energy transfer from an acoustic system to a nonlinear membrane absorber. J. Sound Vib..

[18] Bellet R., Cochelin B., Cote R., Mattei P.-O. (2012). Enhancing the dynamic range of targeted energy transfer in acoustics using several nonlinear membrane absorbers. J. Sound Vib..

[19] Mariani R., Bellizzi S., Cochelin B., Herzog P., Mattei P.-O. (2011). Toward an adjustable nonlinear low frequency acoustic absorber. J. Sound Vib..

[20] Cote R., Pachebat M., Bellizzi S. (2014). Experimental evidence of simultaneous multi-resonance noise reduction using an absorber with essential nonlinearity under two excitation frequencies. J. Sound Vib..

[21] Shao J., Cochelin B. (2014). Theoretical and numerical study of targeted energy transfer inside an acoustic cavity by a non-linear membrane absorber. Int. J. Non-Linear Mech..

[22] Wu X., Shao J., Cochelin B. (2016). Parameters design of a nonlinear membrane absorber applied to 3D acoustic cavity based on targeted energy transfer (TET). Noise Contr. Eng. J..

[23] Wu X., Shao J., Cochelin B. (2016). Study of targeted energy transfer inside three-dimensional acoustic cavity by two nonlinear membrane absorbers and an acoustic mode. ASME J. Vib. Acoust..

[24] Shao J., Wu X., Cochelin B. Study of targeted energy transfer inside 3D acoustic cavity by two nonlinear membrane absorbers. Proceedings of the ASME 2015 International Design Engineering Technical Conferences and Computers and Information in Engineering Conference, IDETC/CIE 2015.

[25] Gendeman O., Starosvetsky Y., Feldman M. (2008). Attractors of harmonically forced linear oscillator with attached nonlinear energy sink I: Description of response regimes. Nonlinear Dyn..

[26] Starosvetsky Y., Gendelman O. (2008). Attractors of harmonically forced linear oscillator with attached nonlinear energy sink II: Optimization of a nonlinear vibration absorber. Nonlinear Dyn..

